# Breast and bowel cancers diagnosed in people ‘too young to have cancer’: A blueprint for research using family and twin studies

**DOI:** 10.1002/gepi.22555

**Published:** 2024-03-19

**Authors:** John L. Hopper, Shuai Li, Robert J. MacInnis, James G. Dowty, Tuong L. Nguyen, Minh Bui, Gillian S. Dite, Vivienne F. C. Esser, Zhoufeng Ye, Enes Makalic, Daniel F. Schmidt, Benjamin Goudey, Karen Alpen, Miroslaw Kapuscinski, Aung Ko Win, Pierre‐Antoine Dugué, Roger L. Milne, Harindra Jayasekara, Jennifer D. Brooks, Sue Malta, Lucas Calais‐Ferreira, Alexander C. Campbell, Jesse T. Young, Tu Nguyen‐Dumont, Joohon Sung, Graham G. Giles, Daniel Buchanan, Ingrid Winship, Mary Beth Terry, Melissa C. Southey, Mark A. Jenkins

**Affiliations:** ^1^ Centre for Epidemiology and Biostatistics, Melbourne School of Population and Global Health The University of Melbourne Carlton Victoria Australia; ^2^ Centre for Cancer Genetic Epidemiology, Department of Public Health and Primary Care University of Cambridge Cambridge UK; ^3^ Murdoch Children's Research Institute Royal Children's Hospital Parkville Victoria Australia; ^4^ Precision Medicine, School of Clinical Sciences at Monash Health Monash University Clayton Victoria Australia; ^5^ Cancer Epidemiology Division Cancer Council Victoria Melbourne Victoria Australia; ^6^ Genetic Technologies Ltd. Fitzroy Victoria Australia; ^7^ Department of Data Science and AI, Faculty of Information Technology Monash University Melbourne Victoria Australia; ^8^ ARC Training Centre in Cognitive Computing for Medical Technologies University of Melbourne Carlton Victoria Australia; ^9^ The Florey Department of Neuroscience and Mental Health The University of Melbourne Parkville Victoria Australia; ^10^ University of Melbourne Centre for Cancer Research Victorian Comprehensive Cancer Centre Melbourne Victoria Australia; ^11^ Genetic Medicine Royal Melbourne Hospital Parkville Victoria Australia; ^12^ Dalla Lana School of Public Health University of Toronto Toronto Ontario Canada; ^13^ Institute for Mental Health Policy Research Centre for Addiction and Mental Health Toronto Ontario Canada; ^14^ Centre for Adolescent Health Murdoch Children's Research Institute Parkville Victoria Australia; ^15^ School of Population and Global Health The University of Western Australia Perth Western Australia Australia; ^16^ Justice Health Group, Curtin School of Population Health Curtin University Perth Western Australia Australia; ^17^ Department of Public Health Sciences, Division of Genome and Health Big Data, Graduate School of Public Health Seoul National University Seoul South Korea; ^18^ Genome Medicine Institute Seoul National University Seoul South Korea; ^19^ Institute of Health and Environment Seoul National University Seoul South Korea; ^20^ Department of Clinical Pathology The University of Melbourne Parkville Victoria Australia; ^21^ Department of Medicine, Royal Melbourne Hospital The University of Melbourne Parkville Victoria Australia; ^22^ Department of Epidemiology, Mailman School of Public Health Columbia University New York New York USA

**Keywords:** breast cancer, causation, colorectal cancer, DEPTH, familial confounding, family data, ICE CRISTAL, ICE FALCON, statistical methods, twin studies, VALID

## Abstract

Young breast and bowel cancers (e.g., those diagnosed before age 40 or 50 years) have far greater morbidity and mortality in terms of years of life lost, and are increasing in incidence, but have been less studied. For breast and bowel cancers, the familial relative risks, and therefore the familial variances in age‐specific log(incidence), are much greater at younger ages, but little of these familial variances has been explained. Studies of families and twins can address questions not easily answered by studies of unrelated individuals alone. We describe existing and emerging family and twin data that can provide special opportunities for discovery. We present designs and statistical analyses, including novel ideas such as the VALID (Variance in Age‐specific Log Incidence Decomposition) model for causes of variation in risk, the DEPTH (DEPendency of association on the number of Top Hits) and other approaches to analyse genome‐wide association study data, and the within‐pair, ICE FALCON (Inference about Causation from Examining FAmiliaL CONfounding) and ICE CRISTAL (Inference about Causation from Examining Changes in Regression coefficients and Innovative STatistical AnaLysis) approaches to causation and familial confounding. Example applications to breast and colorectal cancer are presented. Motivated by the availability of the resources of the Breast and Colon Cancer Family Registries, we also present some ideas for future studies that could be applied to, and compared with, cancers diagnosed at older ages and address the challenges posed by young breast and bowel cancers.

## 1 INTRODUCTION

### 1.1 The problem: Breast and bowel cancers diagnosed in persons ‘too young to have cancer’

Breast and bowel (colorectal) cancers are two of the most common cancers worldwide, with average ages at diagnosis in the 60s. An important minority of these cancers are diagnosed at young ages (e.g., breast cancers diagnosed before age 40 or bowel cancers before age 50) before population screening typically commences, after clinical presentation and typically at a later stage. These young cancers have far greater physical, socioeconomic and psychological impact, have higher mortality and concerningly are increasing in incidence (Acheampong et al., 2020; Araghi et al., 2019; Jayasekara et al., 2019; Kehm et al., 2019; Young et al., 2015). There is a critical need to identify those who are at greatest risk and find out what is causing these malignancies in persons ‘too young to have cancer’ who are, at the moment, unprotected by population screening programmes.

### 1.2 Knowledge gaps

Because they are less common, little is known about the risk factors, causes and outcomes of young cancers. More information on who is at greatest risk is needed to devise cost‐effective programmes for their prevention and early detection. Clearly, prevention and screening must start early in life, and young age is a critical window for cancer prevention. However, epidemiological research on breast and bowel cancer has typically studied people at the ages when most cancers are diagnosed. For example, in case–control studies usually the age of the controls is by design similar to the age at diagnosis of the cases. Cohort studies of breast and bowel cancers rarely recruit young adults; the Breast and Colon Cancer Family Registries (Breast CFR and Colon CFR) are a major exception (see Section 2).

Familial relative risks, and therefore familial variances in risk (see Section 3.1), are much larger at younger ages (Graff et al., 2017; Moller et al., 2016) and therefore provide greater opportunity for discovery of their causes. Yet we still know little. Despite the many advances in genetic research over the last three decades, it is now clear from gene panel testing that high‐risk, pathogenic variants (mutations) in the ‘major’ genes discovered in the mid‐1990s (*BRCA1*, *BRCA2* and the DNA mismatch repair genes *MLH1*, *MSH2*, *MSH6* and *PMS2*) are responsible for only a small proportion of breast and bowel cancers, even of those diagnosed at young ages (see Section 3.2). For carriers of high‐risk mutations in these major genes, there are substantial differences in their risks depending on their family history (Dowty et al., 2013; Kuchenbaecker et al., 2017; Win et al., 2021), the causes of which are not understood. Although known lifestyle factors are associated with modest risk gradients over all ages combined, it is not definitively known which, or even if, they apply to risk of young cancers.

### 1.3 Genetic epidemiology, studies of families and twins, and heritability

Genetic Epidemiology is about how genes and the environment combine to determine disease and health. Studies of families, and especially of twins, have historically been fundamental because they can address questions not easily answered using studies of unrelated individuals alone. This was important before the Human Genome project, when the role of genes on health in general was difficult to establish empirically.

Modern epidemiological analyses of twin and family studies go well beyond the ‘nature versus nurture’ debate captured by the concept of ‘heritability’. Whereas heritability of a continuous trait defined as the proportion of variance caused by genetic factors laid the foundation for variance component analysis, Fisher pointed out that absolute genetic variance was the important parameter, not as a proportion, and referred to its ‘hotch‐potch of a denominator’ (Fisher, 1951). When it comes to risk using binary outcomes under the essentially deterministic liability model, there is not even a finite denominator; total variance in risk is unbounded because perfect risk prediction is not possible.

The concept of ‘heritability’ in terms of ‘percentage of risk explained’ is misleading for guiding genetic research. Under the essentially deterministic liability model, the relationship between the tetrachoric correlation and risk discrimination depends on the disease prevalence (Wray et al., 2010). The more common a disease is, or the older the age at which its prevalence is defined, the higher is its supposed ‘heritability’, despite the fact that familial relative risks decline with age.

Empirical evidence shows that a more realistic view of the way genes and environment combine to determine risk is a multiplicative model of disease incidence, as clearly demonstrated by polygenic risk scores (Mavaddat et al., 2019; Thomas et al., 2020). The maximum variation in genetic risks, and therefore the maximum risk discrimination that can be achieved, is determined by the familial risk ratio for monozygotic (MZ) twin pairs (Hopper et al., 2023).

Moreover, using a measure of a person's underlying familial or genetic risk based on their family data, we found that fundamentally the more a person is at genetic risk, the more important are their nongenetic risk factors—and vice versa (Hopper et al., 2018). That is, genetic risks are not ‘immutable’—much the opposite; there is enormous prospects for reducing (modifying) risk even for people at high genetic risk.

### 1.4 A pathway to new knowledge through the use of cancer family and twin data

In this paper, we describe some existing and emerging family and twin data that can provide special opportunities for discovery. For example, family studies have been the cornerstone for determining cancer risks associated with variants that are rare but likely highly pathogenic, such as protein‐truncating mutations in genes like *BRCA1*, starting with linkage studies of highly selected multiple‐case families (Easton et al., 1995) through to population‐based families sampled irrespective of their family history (Hopper, Southey, et al., 1999). Case‐family designs in which young cases are compared with their cancer‐free siblings are less prone to participation, recall and other biases (Milne et al., 2011). Cancer family studies can have extensive gene panel and family history data to stratify cases and controls (Nguyen‐Dumont et al., 2020). Family designs allow for making inference about causation (Li et al., 2020); see Sections 3.4. and 3.5. Family designs also allow new approaches to be taken, such as consideration of recessive genes (Li, MacInnis, et al., 2022), family‐based genome‐wide association studies (GWAS), and studies of nongenomic familial risk factors such as those for breast cancer being discovered from mammograms (Hopper et al., 2020).

We have identified barriers to discovery, such as a lack of understanding of the existence, utility and power of different family study designs and a lack of awareness of the availability of resources, especially family and twin studies. We also believe from experience that both analyses and translation can benefit from there being close connections between the analytics team and the designers and collectors of the data, and with experts in the content areas. Therefore, we offer thoughts and ideas for how 21st century genetic epidemiology could create a precision population health approach to young breast and bowel cancers, with hopefully much wider implications.

## 2 RESOURCES

We are motivated by existing data and resources, and what might exist in the near future, starting with extraordinary long‐term family‐based cohorts arising from the Breast and Colon CFRs. Both Cancer Family Registries have been funded for a further 5 years and the breast cancer initiative has a focus on recruiting the next‐generation and more young cancer families.

### 2.1 Breast CFR

The Breast CFR is an international cohort study of multigenerational population‐ and clinic‐based families with and without breast cancer funded continuously by the National Institutes of Health (USA) since 1995, conducted in the United States, Canada and Australia (John et al., 2004; Neuhausen et al., 2009; Terry et al., 2016). It comprises 33,000 women and 7000 men from 15,000 families and the 20‐year follow‐up has just been completed. At baseline, we collected blood, biospecimens, medical records, family pedigree, cancer and epidemiology data. There are 1508 *BRCA1* and *BRCA2* mutation carriers, 12,265 breast cancers (8755 diagnosed before age 45) at enrolment with age‐matched population‐ and family‐based controls, 2250 incident breast cancers including 1135 in those unaffected at baseline. There have been comprehensive genetic and environmental risk characterisations.

### 2.2 Colon CFR

Similar to the Breast CFR, the Colon CFR has been funded continuously by the National Institutes of Health (USA) since 1997. Conducted in the United States, Canada and Australasia, it comprises 23,000 women and 19,000 men from 15,000 families and the 20‐year follow‐up has just been completed (Jenkins et al., 2018). At baseline, we collected blood, biospecimens, medical records, family pedigree, cancer and epidemiology data. There are 2300 DNA mismatch repair gene carriers, 12,000 bowel cancers (2000 diagnosed before age 50) with age‐matched population‐ and family‐based controls, 1058 incident bowel cancers including 445 in those unaffected at baseline. This is the world's most comprehensive data and biospecimens for Lynch syndrome and young bowel cancer research.

### 2.3 Harmonisation and future expansions of the Breast CFR and the Colon CFR

The questionnaires, family history, genomic and other data of the Breast CFR and Colon CFR have been harmonised; the baseline questionnaires are designed to overlap. The combined Breast CFR and Colon CFR cohorts cover the full spectrum of risks for both breast and bowel cancers; that is, the Colon CFR includes families without breast cancer and vice versa.

### 2.4 Some population‐complete family and twin cancer data

National genealogies cross‐referenced with health care registers are being increasingly established, starting with the Swedish cancer family database (Yu & Hemminki, 2020) and more recently in Denmark (Athanasiadis et al., 2022). Similarly, algorithms have been developed to identify familial relationships from large emergency health records databases in the United States (Polubriaginof et al., 2018).

There has been a long history of population‐based cancer twin studies, recently combined as the Nordic Twin Study (Harris et al., 2019). The recent FinnGen study in Finland includes genomic data, such as polygenic risk scores (Mars et al., 2022). We have described a number of new analyses, for example, using Inference about Causation from Examining FAmiliaL CONfounding (ICE FALCON) and Inference about Causation from Examining Changes in Regression coefficients and Innovative STatistical Analysis (ICE CRISTAL), that could be performed using this resource (Li & Hopper, 2023; Li et al., 2020, 2024).

The UK Biobank offers the opportunity for family studies given participants who are genetically related can be identified using extensive GWAS data (Brumpton et al., 2020).

## 3 DESIGNS AND NOVEL ANALYSES

There is a long history of statistical methods to analyse family and twin data on disease incidence from a genetic epidemiological viewpoint. Complex segregation analyses based on likelihood theory underpinned linkage studies to identify risk‐associated major loci and estimate penetrance without, and then with, genomic data specific to the loci under consideration. These models have been extended to include polygenic factors (Lange, 2002) and incorporated into risk prediction models such as Breast and Ovarian Analysis of Disease Incidence and Carrier Estimation Algorithm (BOADICEA) (Antoniou et al., 2008; Lee et al., 2019, 2022). A recent example combined multiple measured major genes, in effect discovered families with a very high dominantly inherited risk, which turned out to be due to a known gene, while simultaneously estimating the role of residual familial factors as represented by a factor behaving like a polygenic risk (Li, MacInnis, et al., 2022). Below we present some recently developed approaches to analyse family and genomic data.

### 3.1 Variance in Age‐specific Log Incidence Decomposition (VALID)

Quantifying the familial, genetic, genomic and nonfamilial (environmental) variances in risk is critical for understanding causes and making personalised risk predictions. Under a multiplicative normal risk model for a measured or unmeasured predictor, the variance in risk—with risk defined as the age‐specific log(incidence)—is related solely to the risk gradient on the log incidence scale—which is equal to Δ = the difference between cases and controls in the mean of the predictor—and not to the disease prevalence (Hopper & Carlin, 1992; Hopper et al., 2023). This model refers to risk scores—risk factors transformed, adjusted for age and other confounders and standardised—and is based on the OPERA (Odds PER Adjusted standard deviation) concept that allows risk factors (predictors) to be put into perspective with one another (Hopper, 2015).

As explained in Hopper et al. (2023), there is a simple Unifying Equation that determines the familial risk ratio (FRR) due to a risk score that has a correlation *r* in relatives, namely,

(1) $$\text{FRR}=\text{exp}(r\Delta^{2}).$$

Therefore, there is a maximum to genetic variance given by the FRR for MZ twin pairs, but contrary to what is implied using the liability model and tetrachoric correlation (Wray et al., 2010), there is no natural limit to nongenetic variance. The maximum genetic risk discrimination is dictated by the FRR for MZ twin pairs (FRR_MZ_), alone. Specifically, when *Φ* is the cumulative normal distribution and AUC is the area under the receiver operating curve

(2) $$\text{Maximum}\;\text{AUC}\;=\Phi \left\{{\left[\log\left({\text{FRR}}_{\text{MZ}}\right)/2\right]}^{1/2}\right\}.$$

### 3.2 Application of VALID to breast and bowel cancer

**Figure 1:**
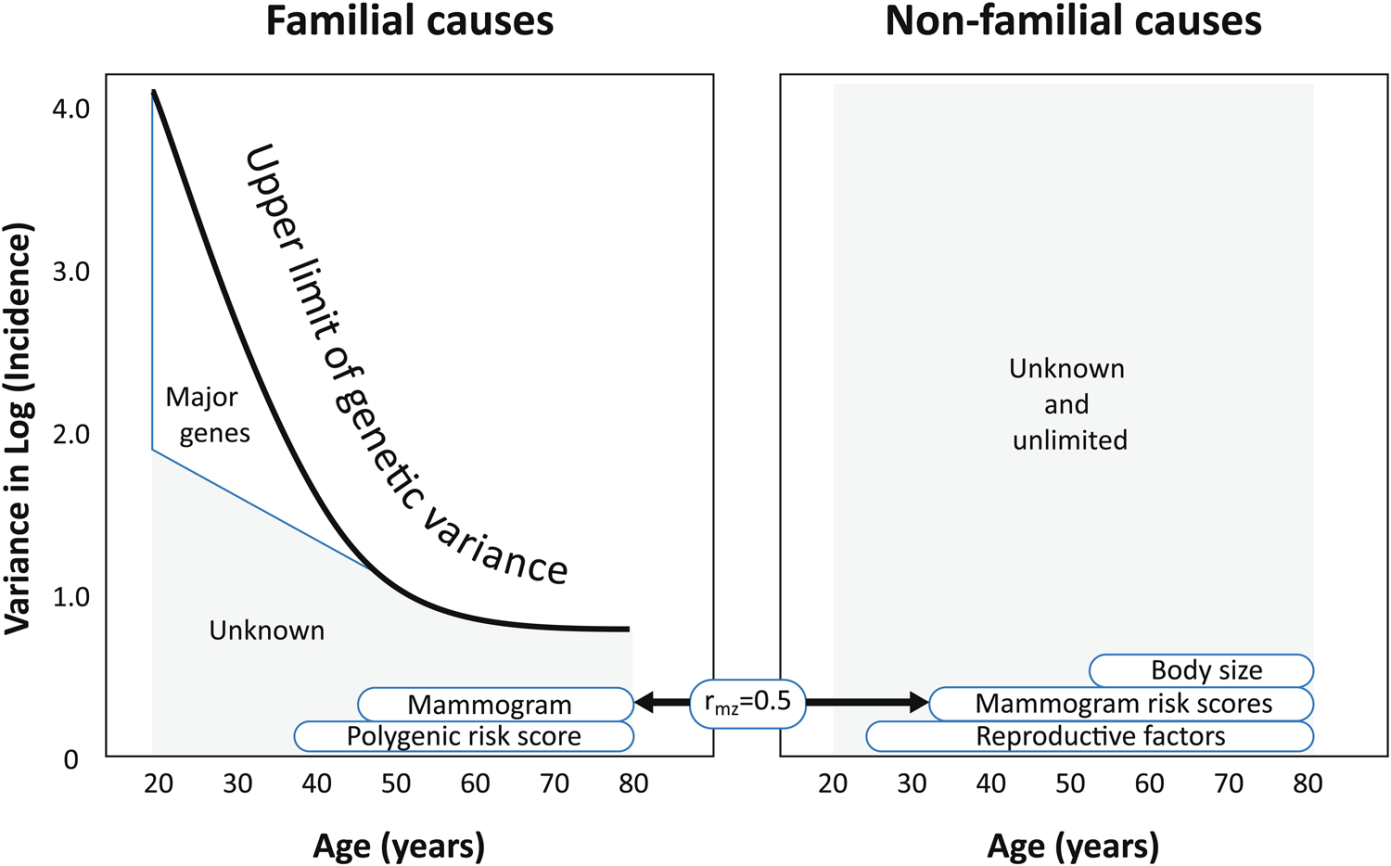
Variance in log(incidence) of breast cancer by age according to familial effects, including rare variants in major genes such as *BRCA1* and *BRCA2*, polygenic risk scores, mammogram risk scores that have a substantial familial component and some other epidemiological risk factors that are mostly nonfamilial, based on the literature (from Hopper et al., 2023).

We applied VALID to breast cancer using the Nordic Twin Study of Breast Cancer summary statistics (Möller et al., 2016), a complex segregation analysis that included our Australian multigenerational family data (Li, MacInnis, et al., 2022), and other publications on breast cancer including our mammogram risk scores (Hopper et al., 2020; Nguyen et al., 2017, 2018, 2020) to decompose the variance in risk as shown in Figure 1. The gaps in knowledge are the shaded regions.

Familial variance is highly age‐dependent, and high‐risk mutations in *BRCA1*, *BRCA2* and other ‘major genes’ explain a proportion at young—but not old—age. There is still a vast amount of variance at a young age, the causes for which are ‘unknown’. In addition, a recent genome‐wide exome sequencing meta‐analysis found that coding variants in other genes explain little variance (Wilcox et al., 2023). The polygenic risk score explains variance at older ages but not at young ages. The mammogram risk scores are correlated by about 0.5 in MZ twin pairs, so contribute to both familial and nonfamilial variance (Nguyen et al., 2022), and are virtually independent to the polygenic risk score (Li, Nguyen, et al., 2022). The epidemiological risk factors explain some variance, but more so at older ages.

**Figure 2:**
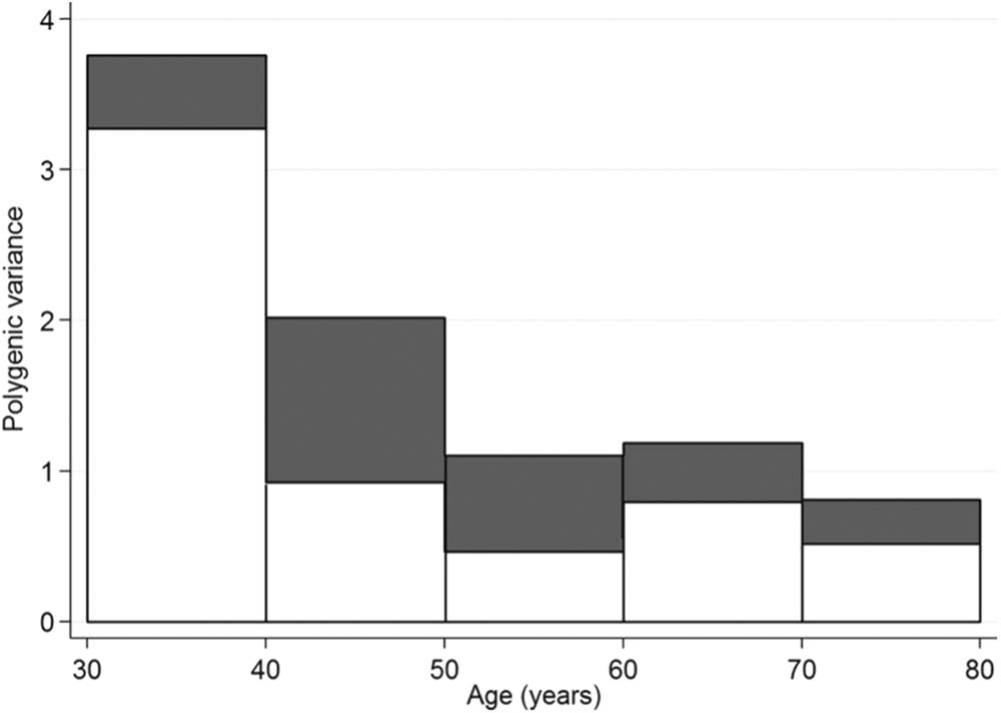
Familial variance explained by hypothetical unidentified major genes (dark grey) and the polygenic component (white) for each 10‐year age group, after excluding families with pathogenic variants in known DNA mismatch repair genes (from Win et al., 2017).

A similar story applies to bowel cancer (see Figure 2), except that: (i) the only major genes of significance are the DNA mismatch repair genes, and they only explain ‘nonpolyposis’ (Lynch syndrome) bowel cancer. The genes implicated in the current polygenic risk scores for bowel cancer in general do not appear to be relevant to Lynch syndrome bowel cancer (Jenkins et al., 2021; Win et al., 2013). There is substantial evidence of other as yet undiscovered major genes for general bowel cancer, as we found from a large segregation analysis (Win et al., 2017). As for breast cancer, the epidemiological risk factors do not explain a substantial amount of variance in risk (Zheng et al., 2020).

### 3.3 DEPendency of association on the number of Top Hits (DEPTH)

We developed the DEPTH method (Makalic et al., 2013) for analysing GWAS data that utilises the power of massively parallel computing to find genomic regions, rather than individual variants, implicated in risk. DEPTH is a discovery tool that can be applied to family data. It uses a sliding window of multiple variants to create a SONAR (Signal‐Over‐Noise Associated with Risk) for each overlapping region that can be interpreted as the log odds in favour of the fitted model. When plotted using a genome browser, the user can easily identify regions of interest. We have applied DEPTH to prostate cancer and identified 112 novel putative risk regions (MacInnis, Schmidt, et al., 2016).

We recently applied DEPTH to bowel cancer data from the Colon CFR (5735 cases and 3688 controls), and 8865 cases and 10,285 controls from other nonfamily studies and compared with 132 published risk regions (Lai et al., 2023). We identified 82 likely risk regions that would not be detected using conventional approaches nor had been identified in previous studies. We found four reproducible risk regions with the strongest signal from the human leucocyte antigens (HLA) region on chromosome 6p21, implicating the immune system in causing bowel cancer.

### 3.4 Within‐sibship comparisons, familial confounding and causation

Within‐sibship comparison designs naturally control for familial (i.e., genetic and nongenetic) factors shared by the siblings, and the extent of these familial confounders will vary across the types of siblings and their ages. However, finding a within‐pair association does not imply the association is causal as it may still be prone to individual‐level confounding, whereas not finding a within‐pair association does not imply the association is not causal. Making inference about causation in the presence of familial confounding can be addressed by the ICE FALCON and ICE CRISTAL methods below (Sections 3.5 and 3.6).

For pairs selected due to being disease discordant, case–control analyses predict risk for controls who, by design, have a family history. Studies of disease‐concordant siblings using the within‐pair difference between ages at diagnosis predict risk for individuals with a strong family history, in that both have a diagnosis. Note that, using a within‐sibship design, inference is being made about exposures of the magnitude of the sample within‐pair differences in exposure, which will likely be less than the differences in exposure across the population for which unmatched studies are making inference. Selection of pairs most discordant for exposures (see e.g., Hopper & Seeman, 1994) will be more powerful per subject and enable more comparable comparisons of within‐ and between‐pairs estimates.

### 3.5 ICE FALCON

Causation is a pivotal issue through its aetiological relevance. It also has implications for prevention and risk reduction given that associations alone are not enough to ‘confer’ or ‘convey’ causation even if the associations are over time, within families or involve a genetic exposure. Randomised controlled trials are expensive, often cannot be conducted for ethical or practical reasons and typically take considerable time leaving the findings vulnerable to being out of date. Therefore, there is a continual reliance on observational data to assess the evidence for causation, especially and even if it is longitudinal.

We developed a powerful way to make inference about causation from family data (ICE FALCON) (Li et al., 2020). The key issues are that ICE FALCON: (i) applies to data on pairs of related individuals who are correlated in their exposure, (ii) fits regression models, estimating within‐individual and across pair coefficients independently and together and (iii) makes inference by comparing the changes in the pairs of regression coefficients.

Briefly, the data are consistent with causation if the cross‐pair coefficient attenuates to the null after including a within‐individual coefficient, but not vice versa. The data are consistent with familial confounding if both the cross‐pair coefficient and within‐individual coefficients attenuate to the same extent after including one another. Reverse causation can be tested in several ways, such as reversing the roles of the presumed cause and outcome in the regression model (Li et al., 2020). These scenarios operate irrespective of individual‐specific confounders.

We have applied ICE FALCON to the atopic march (Hopper et al., 2012), mammographic density (Dite et al., 2008; Stone et al., 2012; Ye et al., 2023), bone architecture (Bui et al., 2013, 2020; Nissen et al., 2023) and DNA methylation (Li et al., 2018, 2019, 2020) and found causal evidence for these exposures. We demonstrated that ICE FALCON can make the same causal conclusion as Mendelian randomisation using much smaller data sets; for example, 66 MZ twin pairs versus 4000 individuals to find that body mass index (BMI) causes DNA methylation, not vice versa (Li et al., 2020). ICE FALCON can be applied to prospective exposure‐outcome data and can be used to test if the tracking of a risk factor with time is causal and therefore justify intervention studies. This issue cannot be readily addressed using Mendelian randomisation as we found for BMI.

### 3.6 ICE CRISTAL

ICE CRISTAL applies to data for unrelated individuals and so is more widely applicable. It is similar to ICE FALCON but replaces the exposure of the relative with the outcome of the relative for the family history (however defined) (Li & Hopper, 2023; Li et al., 2024). In the context of breast and colorectal cancer, ICE CRISTAL considers the extent to which family history is explained (statistically) by including a (familial) risk factor. Again, inference is made by studying the changes in the pair of regression coefficients. Observing that the exposure coefficient does not change after fitting family history, but that the family history coefficient decreases after fitting the exposure coefficient, is consistent with the exposure having a causal effect, as we observed for mammographic density and breast cancer (Martin et al., 2010), and for polygenic risk scores and breast cancer (Li et al., 2024). Familial confounding is consistent with both the exposure and family history coefficients decreasing when estimated together. Statistical inference about the change in a regression coefficient can be made using the methodology attributed to Freedman et al. (1992) that is referenced in Martin et al. (2010).

## 4 POTENTIAL APPLICATIONS

We give some examples of genetic and epidemiological studies that could utilise family or twin data to specifically research breast and bowel cancers being diagnosed at a young age.

### 4.1 To discover factors in early life that likely cause breast or bowel cancer or impact on outcomes for those diagnosed with these cancers

Baseline and follow‐up data from the family and twin cohorts could be used to conduct further case–control and cohort studies, including case–sibling analyses that control for shared familial factors and are less prone to participation bias (Milne et al., 2011). Analyses could focus on early‐life exposures including height, weight at age 18–20, waist‐to‐hip measurements, physical exercise, radiation, use of medications including oral contraceptives and nonsteroid anti‐inflammatory drugs and the reproductive history of females. Creative ways of combining questionnaire data to create new variables with aetiologic relevance, such as the Pike model for the cumulative effect of ovarian hormones (Pike et al., 1983) and reworked to create a risk score for age (Nguyen et al., 2020), could be explored. Interaction terms can be fitted to test if risk associations differ with age at diagnosis or family history (Hopper et al., 2018; Ye et al., 2022).

Timing of exposure could be important. For example, oral contraceptive (OC) use is generally considered to have a short‐term effect on breast cancer risk that dissipates within 5 years of last use. OC use has a protective association with ovarian cancer, so underlying genetic risk is important given the high risk of ovarian cancer for *BRCA1* carriers (MacInnis, Pike, et al., 2016). Using the Breast CFR, we found strong evidence that OC use was associated with a substantial (4.5‐fold; *p* = 0.0001) protective association against breast cancer diagnosed before age 40 for *BRCA1* carriers, but not for *BRCA2* carriers or noncarriers (Milne et al., 2005). The results published to date for mutation carriers are generally null but the literature is almost entirely from studies of women over the age of 40 years; despite *BRCA1* mutation carriers having already reached their peak incidence of 2% per year in their 30s (Kuchenbaecker et al., 2017). Given our gene panel testing that includes cases, controls and relatives younger than age 40 years, these analyses could be revisited with more power and certainty to try to resolve these timing of exposure issues for all risk factors.

**Figure 3:**
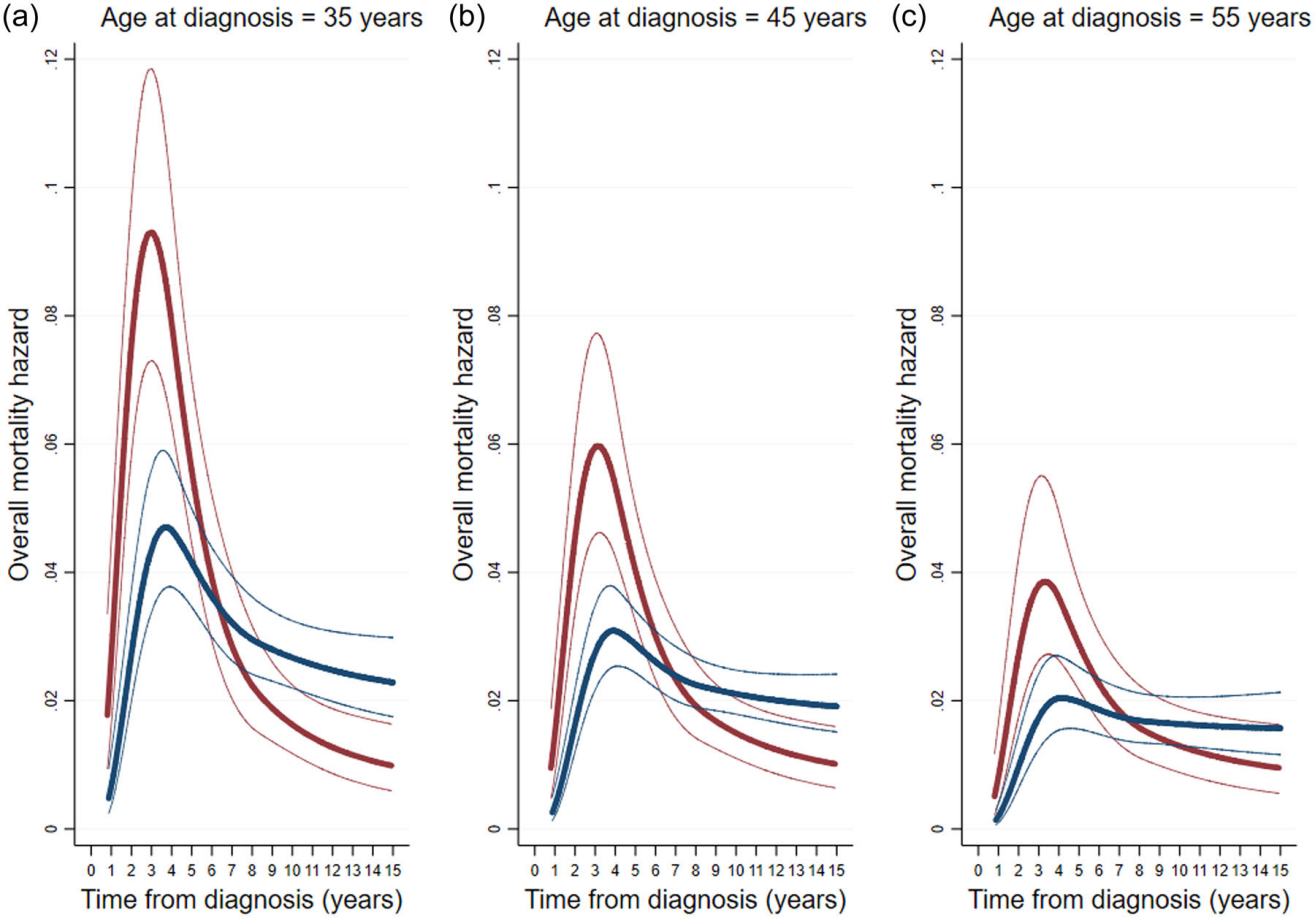
Mortality hazard after breast cancer by time from diagnosis, age at diagnosis, and oestrogen receptor status (negative red, positive blue) with 95% confidence intervals (from Jayasekara et al., 2019). (a) age at diagnosis is 35 years; (b) age at diagnosis is 45 years; (c) age at diagnosis is 55 years.

For outcomes, such as all‐cause or disease‐specific mortality, time since diagnosis would be a natural time scale at least for population‐based incident samples. Figure 3 shows results from fitting flexible parametric survival analyses stratified by type of cancer and age at diagnosis, as we did for oestrogen receptor status and mortality following breast cancer (Jayasekara et al., 2019). Interaction terms for age at diagnosis could be fitted to quantify the extent to which associations depend on age at diagnosis and other measured covariates.

Opposite‐sex twin pairs allow for within‐pair analyses that address sex differences by controlling for age, on average 50% of autosomal genes and shared environment including shared placenta in utero. This avenue of investigation could be informative for bowel cancer.

### 4.2 To identify new genetic causes and modifiers of risk for breast and bowel cancers

DEPTH (see Section 3.3) and other new analytic techniques could be systematically used to analyse the GWAS data of the Breast Cancer Association Consortium of 137,000 cases and 119,000 controls (Michailidou et al., 2017) and the Colon CFR and associated bowel cancer studies (COloRECTal Cancer Transdisciplinary study/Genetic Epidemiology of Colorectal Cancer cOhort [CORECT/GECCO]), which currently comprise 60,000 bowel cancers and 70,000 controls (Cheng et al., 2014; Lai et al., 2023; Mahmood et al., 2023). We have developed and run a pipeline to harmonise the different genome‐wide array data across the world's major breast and bowel cancer GWASs to the Human Genome Version 19 (hg19/GRCh37 genome build). Findings could be integrated to allow biological contextualisation using, for example, Ingenuity Pathway Analysis software (Kramer et al., 2014). Candidate genes could be subjected to Core Analysis to provide a measure of the overlap between each gene set and three Gene Ontology terms: Molecular Function, Biological Process and Canonical.

### 4.3 To estimate penetrance for genes and variants as a function of characteristics

As genetic technologies advance and more genes and genetic regions are implicated in cancer risks, estimating penetrance (age‐specific incidences) associated with having specific variants or classes of typically rare variants (such as those currently classified as being of ‘unknown significance’, will become more important. Pedigree analyses of families, once properly adjusted for their ascertainment (Li, Silvestri, et al., 2022; Win et al., 2021), can be used to estimate penetrance as a function of a variant's supposed pathogenicity predicted by different classification methods (e.g., Align‐GVGD, Polyphen, Grantham matrix scores and sequence conservation in mammalian species) (Dowty et al., 2014). Quantitative estimates can then be attributed to a priori clinical categorisations, such as ‘pathogenic’ or ‘likely pathogenic’ and used to help better define, and interpret, clinical classifications.

### 4.4 To determine how known and unknown genetic and other factors combine to determine, or cause, risk of breast and bowel cancers

Multivariable multiplicative logistic and Cox regression models could be fitted to determine how risk factors combine; lack of interactions on the multiplicative scale implies interactions on the absolute scale. Gene–environment interactions can be assessed by fitting interaction terms for measures of underlying familial risk profile based on pedigree models (Hopper et al., 2018) such as BOADICEA for breast cancer (Antoniou et al., 2008; Lee et al., 2019, 2022) and CRISP (Colorectal cancer RISk Prediction decision support tool) for bowel cancer (Emery et al., 2023; Walker et al., 2017, 2018). ICE FALCON could be applied to longitudinal data from siblings and twin pairs to assess if a risk factor at a young age has a causal effect on that risk factor at a later age, and if it has a casual effect on the cancers, information critical for informing prevention.

These family analyses also give insights into the role of familial confounding. For example, we found that height was a risk factor for breast cancer when using population controls but not when using siblings as controls (Milne et al., 2011). This implies that a substantial proportion of the height association is due to familial factors that cause both height and breast cancer. ICE FALCON analyses could further clarify interpretation of this observation.

### 4.5 Qualitative research

Studies of families and twins allow for comparisons and observations on one another that could give insights not otherwise possible, with special relevance to early life exposures. For example, case–control questionnaire studies have asked female twin pairs to compare one another at the time of puberty using questions such as: who went through puberty first, who was tallest, heaviest and whose breast developed earlier (Hamilton & Mack, 2003; Swerdlow, 2002). Further exploration, using qualitative in‐depth interviews of participants' experiences, could contribute additional insights and clarify details that questionnaire studies could not hope to answer. Qualitative studies of relative pairs discordant for cancer, for instance, could generate hypotheses about potential early life causes, as semistructured in‐depth interviews allow participants to raise issues and experiences that are not necessarily apparent to researchers. Qualitative studies of relative pairs discordant for exposures could also provide more reliable evidence on potential causes, with implications for prevention. For example, we previously found evidence from statistical modelling using the regressive logistic model developed by the late George Bonney that a woman's smoking status was strongly predicted by the smoking status of her next oldest sister (Hopper, Jenkins, et al., 1999). We are currently conducting a twin study of breast and colorectal cancer screening practices with the intention of conducting qualitative research with twin pairs who differ in screening practice to help explore the reasons for their discordancy further.

## 5 DISCUSSION

This paper presents some ideas on how the gaps in knowledge about the genetic and nongenetic causes of cancers, especially breast and colorectal cancers diagnosed at young adult ages for which impact is greatest yet least is known.

For example, even though having a family history is a much stronger predictor of young disease, the vast majority of these families do not have a known cause despite the latest gene panel testing. There are ‘major’ genes for young breast and bowel cancers yet to be discovered. Current polygenic risk scores are not making a big difference, especially for those at a young age for whom screening might not be available. There is no established polygenic risk score for Lynch syndrome (the main familial nonpolyposis bowel cancer) despite wide variation in familial risk. Known nongenetic risk factors explain little variation in risk and risk discrimination on a population scale.

Advances in genetic, epigenetic, proteomic and other technologies offer great potential. Family and twin study designs are innovative and powerful, and extensive resources are already available and being enhanced, and it is important that they be extensively used. The combination of new technologies, powerful analytic tools for twin and family designs and extensive resources could provide exciting opportunities for new discoveries. It is also important that the expertise and experiences of those involved in creating and sustaining long‐term studies and experience in the design and statistical analyses are utilised to avoid errors and guide interpretation.

### 5.1 Limitations and future possibilities

Genetic Epidemiology was initially defined as ‘a science which deals with the aetiology, distribution, and control of disease in groups of relatives and with inherited causes of disease in populations’ (Morton, 1982) and traditionally based on studies of genetically or otherwise related individuals. As well as its uniqueness and strength, this familial aspect has posed limitations on research and made it more difficult to replicate findings. Collecting family data, especially on a large scale and with the epidemiological requirements of defined sampling with high responses from probands and relatives alike, is more complicated than studying isolated individuals alone. We were able to address these limitations through dedicated resource‐building funding from the National Institute of Health specifically to study breast and colorectal cancers. With advances in genetic technologies, large‐scale genotyping of research studies is being achieved and in doing so related participants are being accurately identified, thereby opening up new opportunities for family‐based studies and analyses; see Section 2.4.

We hope that this article will attract others to join us in addressing the challenges and opportunities we have identified.

## CONFLICT OF INTEREST STATEMENT

G. S. D. is an employee of the Genetic Technologies Ltd. The other authors declare no conflict of interest.

## Data Availability

Data sharing is not applicable to this article as no new data were created or analysed in this study.
